# Comparison of Pedestrian Detectors for LiDAR Sensor Trained on Custom Synthetic, Real and Mixed Datasets

**DOI:** 10.3390/s22187014

**Published:** 2022-09-16

**Authors:** Paweł Jabłoński, Joanna Iwaniec, Wojciech Zabierowski

**Affiliations:** 1 Department of Robotics and Mechatronics, Faculty of Mechanical Engineering and Robotics, AGH University of Science and Technology, Mickiewicz Alley 30, 30-059 Cracow, Poland; 2 Department of Microelectronics and Computer Science, Lodz University of Technology, ul. Wólczańska 221, 93-005 Łódź, Poland

**Keywords:** LiDAR, ADAS, deep learning, object detection, Carla simulator, artificial data, Waymo open dataset, YOLOv4

## Abstract

Deep learning algorithms for object detection used in autonomous vehicles require a huge amount of labeled data. Data collecting and labeling is time consuming and, most importantly, in most cases useful only for a single specific sensor application. Therefore, in the course of the research which is presented in this paper, the LiDAR pedestrian detection algorithm was trained on synthetically generated data and mixed (real and synthetic) datasets. The road environment was simulated with the application of the 3D rendering Carla engine, while the data for analysis were obtained from the LiDAR sensor model. In the proposed approach, the data generated by the simulator are automatically labeled, reshaped into range images and used as training data for a deep learning algorithm. Real data from Waymo open dataset are used to validate the performance of detectors trained on synthetic, real and mixed datasets. YOLOv4 neural network architecture is used for pedestrian detection from the LiDAR data. The goal of this paper is to verify if the synthetically generated data can improve the detector’s performance. Presented results prove that the YOLOv4 model trained on a custom mixed dataset achieved an increase in precision and recall of a few percent, giving an *F*1-score of 0.84.

## 1. Introduction

Nowadays, the automotive industry puts a lot of research effort into the development of autonomous driving systems for vehicles, due to the intensification of promising results in the field of machine learning technologies used for object detection in recent years. Additionally, sensors used for advanced driving assistance systems (ADAS), such as cameras, radars or LiDARs, increase performance in terms of data quality and reliability. Algorithms used in most of the present systems are based on the supervised learning models, which means that the performance of such algorithms is highly correlated with the size and quality of the dataset used for the training process. The problem is that data gathering and the following process of object labeling are costly and time-consuming. What is even more frustrating, in the case of upgrading sensor capabilities, is that reuse of already created datasets is not efficient and, consequently, the new sensor setup usually requires totally new datasets. Therefore, the idea of making use of high-performance computer technologies and generating synthetic data with already labeled objects is highly appealing. Such an approach can significantly increase the performance of algorithms and, at the same time, decrease the expenses on projects.

The biggest challenge in terms of synthetic data generation is to provide computer simulation where sensor models and simulated environments provide similar data quality as the real hardware installed in cars. For this task, one of the most beneficial advances is the achievements of computer 3D rendering technologies developed mostly for the computer games industry, such as Unity engine or Unreal engine. These high-fidelity and high-quality rendering engines are capable of simulating the road environment with traffic. Additionally, there are available programming environments such as Carla [1], where not only the road environment but also sensor models can be simulated.

The main goal of this paper is to answer the question of whether it is possible to improve the LiDAR sensor’s pedestrian detection performance with the use of synthetically generated data? The root cause of this question is the phenomenon known as “synthetic-to-real gap” [2]. In most cases, a huge gap between the performance of the detector working on real and synthetic data appears. The challenge is to create synthetic data as good as real to bridge the gap.

In this paper, attention is paid to the problem of detection of pedestrians, who are the most vulnerable and valuable traffic participants. They are smaller in comparison to the vehicles, which makes it more challenging to correctly detect this kind of object. In the proposed approach, LiDAR point cloud is generated automatically from the road scenarios where pedestrians are present. In the course of the carried-out research, these input data were used to train the YOLOv4 neural network architecture to perform pedestrian detection. It is important to highlight that all detectors trained for the research were validated on the real sensor data. The Waymo [3,4] open dataset was used as the source of the real LiDAR data.

The main advantage of LiDARs is that data generated by the sensor represent its surroundings with high precision in terms of distance to the objects. Neither camera nor radar can estimate the distance to the object with such a high precision. LiDARs have been mostly used as ground truth data sources for the camera of radar sensor algorithms. This situation is beginning to change due to the decreasing cost of LiDAR sensors. Nowadays, increasingly more autonomous applications use LiDARs [5,6,7,8]. A good example of a LiDAR sensor application is the Waymo project. Waymo vehicles are equipped with multiple sensors around the car. Five of them are cameras and, additionally, there are five LiDARs, four short-range and one mounted on the vehicle roof. The LiDAR on the top of the Waymo vehicle is considered as the source of the real data for the experiments presented in this paper. Carla sensor simulation is set up in such a way as to mimic the data generated by the Waymo top LiDAR. Experimental results presented in this paper define the performance of pedestrian detection algorithms, which are trained on real, synthetic and mixed data. Mixed data used in the course of the research included real and synthetic data in two variants of proportions. Around 10 k frames were generated and used for the experimental detector training.

The structure of the paper is as follows. In the first section, the issues related to the development of autonomous driving systems for vehicles and object detection methods are introduced. The state of the art is briefly presented in Section 2. The third and fourth sections are dedicated to the proposed methodology and methods used in the research presented in the paper. The carried-out experiments are described in Section 5. The obtained results are presented and discussed in Section 6. Finally, the paper is concluded in Section 7.

## 2. Related Works

Recently, the problem of synthetic data generation has generated great interest in the research community due to its possible advantages in comparison to real data gathering. Plenty of publications tackle this topic, with different technologies and methodologies applied. Many papers such as [2,9,10] focus on synthetic camera images as a basis for object detection of various types of objects. In the case of color images, unlike in LiDAR sensor measurements, light and weather conditions have a significant influence on the results of object detection. That is why different sensors require different methodologies in the case of synthetic data generation. There are reported examples of experiments which use synthetic LiDAR data for deep neural network training, e.g., in [9,11,12]. All of these papers describe the 3D object detectors trained on the raw LiDAR point clouds. Such a method requires a much more complicated topology of a deep neural network than the one proposed in this paper, where the range image [13,14] is the input for the algorithm. These differences are discussed in more detail in Section 4.3. It should be stressed that, in the mentioned papers [11,12], the older versions of Carla with much simpler collision boxes were used, which resulted in huge differences between the real and synthetic point cloud data. Section 4.2 of the paper shows the difference between the old and new versions of the Carla LiDAR model.

Apart from different data generation methodologies, different rendering engines can be used to prepare the road scenarios. For instance, in [2,9], GTA V with a Script Hook V library was used. In [15], the researchers used the Synthia dataset generated by the Unity platform, which is a direct concurrent to the Unreal Engine. For the experiment proposed in this paper, the Carla simulator was chosen. In comparison to GTA V or Synthia, it is provided with a highly configurable LiDAR model. That is why Carla was selected to generate realistic synthetic LiDAR sensor data that can mimic the characteristics of the real LiDAR point cloud. All mentioned rendering engines use the ray casting method to calculate the point cloud from the simulation scenario. The same method is used by Carla.

## 3. Methodology and Methods

In the course of the carried-out research, the LiDAR pedestrian detection algorithm was trained on the real, synthetic and mixed data. Synthetic data are generated by the LiDAR sensor model in the 3D Carla (**C**ar **L**earning to **A**ct) simulator and the real data are taken from the Waymo open dataset [3]. Carla is an open-source simulator developed to support training, prototyping and validation of autonomous driving systems. Carla is a layer of the Unreal Engine 4 (UE4). The UE4 rendering engine provides state-of-the-art rendering quality, allowing us to perform and generate a realistic road environment [16]. Carla allows us to configure a range of suitable sensors to be simulated, starting from the basic singular camera simulations to high-complexity setups of LiDARs, radars and cameras. Additionally, Carla supports the simulation of complex, high-quality weather conditions, such as rain, wind, fog, cloudiness and more. Unfortunately, those weather conditions do not affect LiDAR sensor data generation. Especially relevant for the LiDAR sensor model would be rain.

### Pedestrian Detection in Autonomous Driving Systems for Vehicles

Nowadays, the vast majority of artificial intelligence-based object detectors used in the automotive industry are suitable for recommendation systems only [17]. Such systems aim at providing recommendations and warnings that support decision making by the human driver. The type of neural model for such applications depends heavily on the requirements concerning accuracy and latency, i.e., the time taken to process one unit of data, under the assumption that only one unit of data is processed at a time [18]. For instance, in the parking systems searching for free spaces, slow accurate models are usually used, while car collision warnings are provided by fast but inaccurate models [17].

Recently, great attention has been paid to the possibilities of improving the accuracy of real-time object detectors, which would enable their usage in autonomous driving systems for vehicles. In the field of the automotive industry, the task of real-time object detecting is especially important in relation to pedestrians, since they are the most vulnerable participants in road traffic [19,20]. The numerous attempts to apply deep neural networks to pedestrian detection on the basis of visual information of different forms are reported in the literature [10,21,22,23]. However, the high latency of the proposed neural models and the required high object detection accuracy, combined with the limited capabilities (processing power) of GPUs used in autonomous vehicles, often prevent these systems from industrial deployment. The intensification of the research into pedestrian detection for autonomous systems that has been observed recently resulted from the development of the consecutive versions of the YOLO algorithms [17,24,25]. Unique features of these algorithms (especially YOLOv4) include the high speed of computations and the high accuracy of the obtained results, combined with the excellent learning capabilities, making these algorithms a promising choice for autonomous vehicles.

In the research, the results of which are presented in this paper, the YOLOv4 algorithm was used for the real-time pedestrian detection task performed for the real, synthetic and mixed datasets.

## 4. LiDAR Sensor Data

The LiDAR sensor operation is based on the measurement of the time of flight of the light. LiDAR emits a short light pulse, which reflects from surfaces and is received back by the sensor. Distance to any object is estimated based on the time of flight of the light pulse emitted by the sensor. The newest LiDARs use a multichannel rotary core to scan the environment around the sensor. A higher level of channels gives a denser point cloud on the sensor output.

### 4.1. LiDAR Sensor Data Provided by Waymo Open Dataset

**Figure 1:**
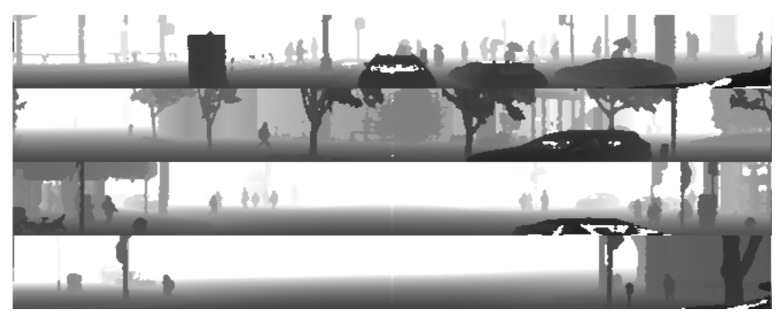
Example frames of range images collected from the Waymo dataset.

The dataset provided by the Waymo company is an open-source dataset which includes recordings out of over 6 h of driving, covering an area of 76 km^2^. All sensor recordings are stored in the packages of around 200 frames. Files are packed into the *tfrecord* format to compress the size of the data to be downloaded from the Waymo repository. To read out the dataset frames, the Python environment with the TensorFlow package was used. The Python environment allows us to convert the top LiDAR point cloud into the range images. Example range images from the Waymo dataset are depicted in Figure 1. The original image includes a 360-degree view, which, in the course of the experiments, was cropped by half to 180 degrees, in order to consider only the view to the front of the vehicle.

**Figure 2:**
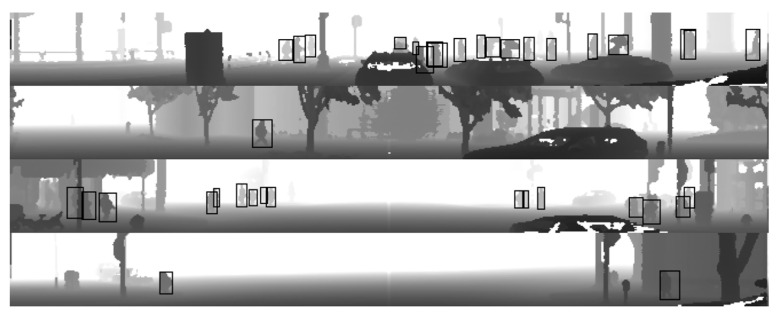
Example frames of range images collected from the Waymo dataset with bounding boxes drawn.

*Tfrecord* files also include bounding boxes for the objects, such as vehicles, pedestrians, cyclists and others, which are synchronized with the sensor frames. For the LiDAR sensor, 3D bounding boxes are also defined. 3D bounding boxes were not used for the experiments, since the input format was downgraded to 2D. All 3D LiDAR bounding boxes were projected into 2D object boxes and fit into the size of the range images. Figure 2 depicts the same frames as Figure 1, but with bounding boxes drawn on pedestrians. Data prepared from the Waymo dataset were used as real input data for pedestrian detector algorithm training.

What is important is that only the real data were used to validate the detector performance both for the real and synthetic training datasets.

### 4.2. LiDAR Sensor Data Generated by Carla Simulation

**Figure 3:**
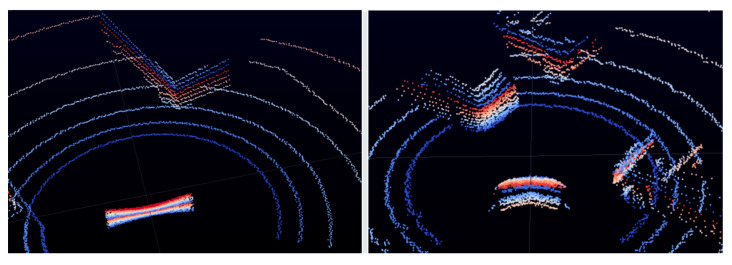
Comparison of LiDAR representations of cars in Carla 0.9.9 (**left**) and Carla 0.9.12 (**right**).

The Carla simulator uses the ray-casting ability of UE4 with realistic 3D collision objects to generate synthetic LiDAR sensor data from the road environment. Carla allows us to configure the sensor’s general parameters, such as a field of view (*FOV*), number of channels, rotation speed, number of generated points per second and others. LiDAR data used in this paper for the training of the YOLOv4 net were generated by Carla version 0.9.12. It is worth mentioning that the older versions of Carla (<0.9.9) provided much simpler representations of 3D object collision boxes used by ray casting (Figure 3). That is why, while using the version 0.9.12, LiDAR data are much more useful and more like the real LiDAR data.

The purpose of the research presented in this paper was to experiment with synthetic LiDAR data, which are further used together with the real sensor data to extend dataset training and to compare the performance of the detector with the one trained on the real data only. That is why the simulated LiDAR parameters were set similarly to the parameters of the Waymo top LiDAR sensor [3]. Such an approach allows the fitting of synthetic data to the Waymo real data. Table 1 presents the comparison of Carla LiDAR parameters to the Waymo top LiDAR parameters.

Some parameters of a real LiDAR sensor cannot be set directly in the simulation model. For instance, the number of generated points per second (*P_ps_*) results from the values of other LiDAR parameters. For the assumed values of LiDAR parameters (Table 1), the number of points generated by the sensor per second (*P_ps_*) can be calculated as follows:

(1) $$P_{ps}=\frac{FOE_{H}*N*f}{\varphi_{H}}=>\frac{360˚*64*5\;\mathrm{Hz}}{0.1358˚}=848306\;\frac{1}{s}$$

where $FOE_{H}$ denotes the horizontal field of view [˚], N the number of channels, f the rotation speed [Hz] and $\varphi_{H}$ the horizontal angular resolution [˚].

The parameters of rotation rate and points per second generated for the Waymo sensor are not defined precisely by the company. That is why the Carla parameters were estimated in such a way as to generate the range image of the same size as the one provided by the Waymo dataset.

The Carla environment can generate a perfect cloud of LiDAR points gathered from the sensor’s surroundings. This perfection cannot be realized by the real sensor due to many distortions existing in the real environment. That is why the Carla LiDAR model allows us to manipulate additional parameters to mimic a real imperfect point cloud. Table 2 describes five of those parameters.

**Figure 4:**
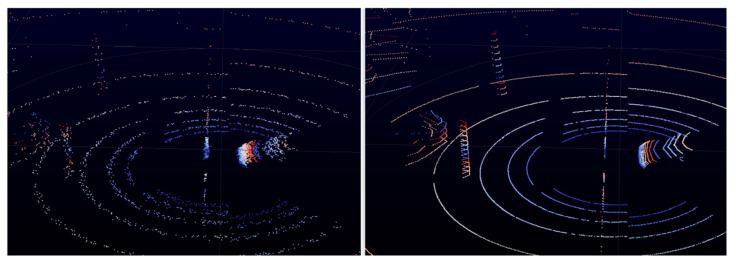
Comparison of the simulation point cloud with and without additional model parameters used. Both images depict the same road scenario.

Properly selected values of parameters described in Table 2 make it possible to mimic the randomness of the real sensor data. For example, the datasheet of Velodyne LiDAR HDL-64E [26] defines the range precision as two centimeters. By setting the noise standard deviation parameter to 0.02 m, the simulated model mimics this range precision. Figure 4 depicts the differences between the data generated with and without the use of additional parameters.

Figure 4 clearly visualizes how perfect the points are in the right picture in comparison to the left picture, where points are randomized and noisy. These simulation capabilities make simulated data much closer to the real sensor data [27].

### 4.3. Synthetic LiDAR Data Preparation for the Training Dataset

The generated data include three-dimensional space interpretation. That is why the standard approach to LiDAR point cloud data used in machine learning is to train the network to detect and predict the position of the necessary objects in the three-dimensional space. The input for the considered neural network has the form of a set of 3D points and the output consists of 3D bounding boxes of detected objects. This straightforward solution has one major disadvantage, which is an additional dimension, in comparison to the well-known 2D image object detection problem. It extends the complexity of the neural network model and decreases detection speed of the trained model. There are solutions where 2D projection BEV—bird’s-eye view—is used [28]. BEV decreases the point cloud row data dimension to 2, but this solution has the disadvantage of information loss. BEV data are flattened, so there is a loss of the object height information in this method. The idea presented in this paper is to simplify the problem to two dimensions, without losing precise information in all dimensions. This approach is known as the range image conversion [13,14].

**Figure 5:**
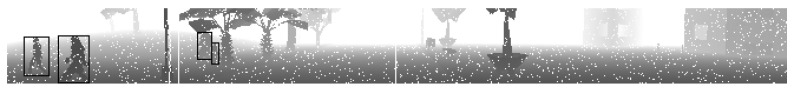
Example raw range image generated from the one-point cloud frame. The image includes four labeled pedestrians where one is a child.

The data generated by simulation were converted to range images before they were used for machine learning. Conversion starts from image size definition. The goal is to generate the same image as the one provided by the Waymo open dataset. The simulated LiDAR generates a vertical view in 64 channels, so the image height was defined as 64 pixels. The simulated sensor’s horizontal *FOV* is 360 degrees and the horizontal angular resolution is set to 0.1358 degrees, so the initial image amounts to 1324 pixels. To decrease the image width in comparison to height, the horizontal *FOV* of the range images was decreased to 180 degrees of the front view. After that change, the image size was defined as 64 × 662 pixels. An example image of one point cloud frame is depicted in Figure 5.

**Figure 6:**
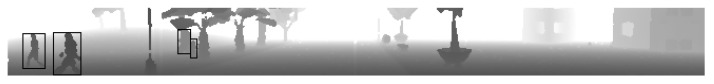
Example range image generated from the one-point cloud frame after the median blur operation was performed.

Figure 5 depicts a point cloud with four pedestrians labeled. Not all pedestrians are clearly visible in this image, mostly due to its graininess. Each pixel of the image represents a particular part of the sensor’s *FOV*, but the sensor does not create all of the points (see Figure 4). It means that all white dots on the picture represent missing points from the sensor measurements. In order to remove missing white points from the image, the median blur operation with a kernel size of 3 was performed. Median blur removes noise and increases the visibility of edges (Figure 6).

**Figure 7:**
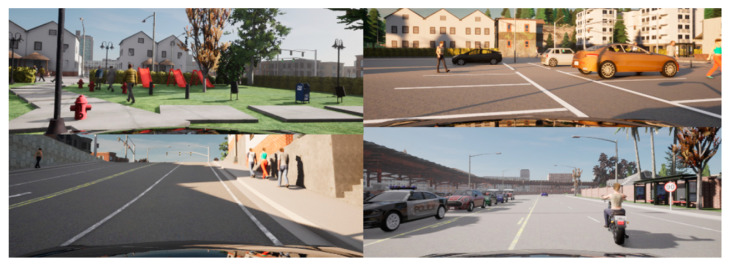
Visualization of example scenarios created for LiDAR data recordings.

The first step of the research consisted in setting up the simulation sensor correctly to obtain data deceptively reminiscent to the real Waymo data. The second step was to generate images including a variety of road scenarios with pedestrians. For this purpose, randomized traffic scenarios including cars, pedestrians, bikes and cyclists were set up in the Carla simulator. LiDAR data generation was performed on seven different maps available by default in Carla. Examples of prepared scenarios are depicted in Figure 7.

Prepared scenarios included frames with none up to a dozen visible pedestrians. It is important to say that maps are only the placeholder for the traffic, which is randomized in Carla. This means that actors such as pedestrians or vehicles are moving around the simulated city all the time. None of the data saved for the experiment from the LiDAR sensor are the same; all the situations are randomly generated by the simulator. Only the start point for actors can be the same. With such a design of the recorded scenarios, pedestrians are scanned in a variety positions, standing, walking, from all the angles around them.

### 4.4. Automatic Labeling for Synthetic LiDAR Data

**Figure 8:**
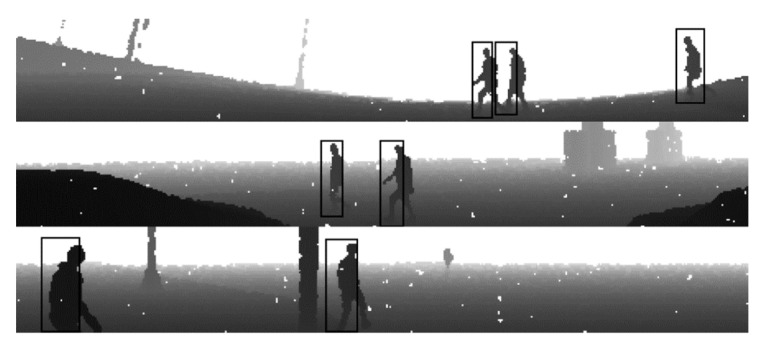
Examples of mismatched labels generated by simulation.

The most beneficial feature of the synthetic data method regarding usability in machine learning is that the generated data are already labeled. The object of interest is already located in the image. This removes one of the most expensive and time-consuming tasks of object labeling in the process of data preparation for machine learning. The initial idea in the case of the Carla simulator and LiDAR data was to calculate the labels of objects from the position of each actor in the simulated world. The bounding box projection of the pedestrian is calculated for each lidar point cloud at the same time as data are saved. This solution for automatic labeling turned out to be imprecise. The examples of mismatched labels are depicted in Figure 8.

Some of the labels miss the pedestrians or do not cover them fully. The root cause of such errors lies in the simulation multithreading process. One thread is responsible for generating the LiDAR sensor point cloud and the other one calculates the position of the pedestrians. LiDAR point cloud generation requires vast amounts of computational resources. That is why a significant delay appears between the simulation threads. The faster the pedestrian or ego vehicle moves, the bigger the mismatches. This labeling issue significantly affects the results of the trained pedestrian detector. That is why the labeling process had to be improved.

**Figure 9:**
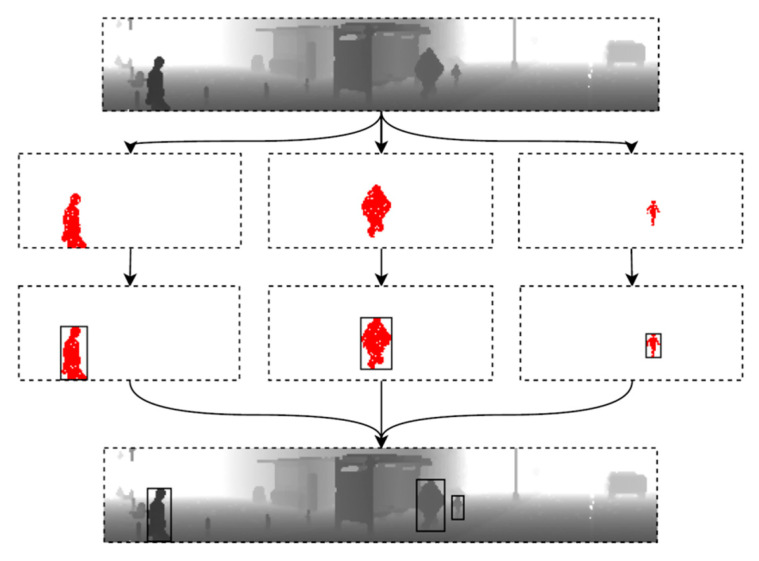
Automatic labeling process with the use of semantic LiDAR data tags.

New versions of Carla give, out of the box, an additional sensor model called segmentation LiDAR. This sensor generates the same data as the standard LiDAR, but within the ray-casting process, it marks each reflected point with a segmentation tag and unique identifier. The segmentation LiDAR point cloud includes additional information about the reflected surface, such as whether it is a vehicle, pedestrian, road, vegetation or something else. This new way of object labeling calculation uses segmentation information from the LiDAR point cloud to label pedestrians precisely. While the range image is generated, each point is colored with a pedestrian tag. In the next step, the minimal rectangle around the colored pixels is calculated. That is how the precise pedestrian label is obtained. The process of label calculation is depicted in Figure 9.

This process can be performed in order to automatically label and tag objects from the simulation. Sometimes, it happens that the tagged object is only partially visible or only a small part of the shape is visible, mostly because it is obscured by other objects. In such a case, really small labels are filtered out and are not considered in the training process.

## 5. Experiments

### 5.1. YOLOv4 Algorithm

Under the term of pedestrian detection, a specific application of the object detection problem is understood. Its importance has grown in recent years due to the increasing interest in the development of autonomous driving systems for vehicles [29]. Evolution of object detection methods and, subsequently, pedestrian detection methods can be divided into two main periods: the classical or traditional object detection period and the period of deep learning-based methods.

The period of pedestrian detection based on deep learning goes back to 2014, when the conference paper by Since Girshick et al. [30] was presented and published. In the literature, all the deep-learning detection methods are divided into two categories: two-stage processing methods and one-stage processing methods, respectively [31]. In the case of the two-stage processing methods, “suggestion” boxes for possible objects are generated first. In the second step, the “suggestion” boxes determined in the first step are used as a basis for further predictions. Contrary to the two-stage methods, one-stage processing methods provide the object area with the feature map and the final prediction result directly. In 2015, the first single-stage YOLO detector was proposed [31], which, due to its algorithmic simplicity and high-speed detection capabilities, was treated as a milestone in the history of pedestrian detection method development. In the later versions of YOLO algorithms—YOLOv2 [32] and YOLOv3 [33]—detection accuracy has greatly improved in comparison to YOLOv1 and a balance between speed of computation and accuracy has been obtained. In 2020, Bochkovskiy proposed YOLOv4 [34], which, due to real-time performance, high accuracy of the obtained results and excellent learning capabilities [35,36], is a promising choice for autonomous vehicles.

### 5.2. Evaluation Metrices

Performance evaluation of any algorithm is essential to understand if the model is optimally developed. Therefore, there exist many evaluation metrics that are used for assessing the quality of the model’s output [37].

In the case of the research discussed in this paper, the algorithm’s output is a bounding box surrounding the pedestrian (person) in the image. Therefore, model performance can be evaluated in two steps. First, it is important to check if the model has correctly detected the required class of objects, so the people are present in the analyzed images. Secondly, the accuracy of determining the bounding box surrounding detected objects of the considered class has to be evaluated.

**Figure 10:**
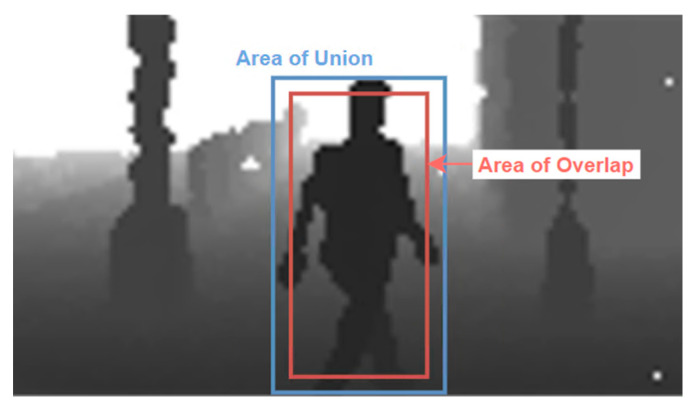
Intersection over Union example.

The metrices proposed in the literature are based on the number of True Positives (TP), False Positives (FP), True Negatives (TN) and False Negatives (FN) in a confusion matrix [38,39]. Assessment of to which of these categories the prediction belongs is based on the value of the **I**ntersection over **U**nion (IoU) measure:

(2) $$IoU=\frac{area\;of\;overlap}{area\;of\;union}\;$$

which determines the amount of overlap of the generated bounding box and the ground-truth bounding box (Figure 10). The values of the IoU belong to the interval <0,1>. If two boxes do not intersect, the IoU equals 0. On the contrary, if they completely overlap, the areas of intersection and the union areas are equal and, consequently, the IoU equals 1.

By calculating the IoU, we can assess whether the output of detection is valid (TP) or not (FP):

- True Positive (TP): correct detection, IoU ≥ threshold,
- False Positive (FP): wrong detection, IoU < threshold,
- False Negative (FN): ground-truth bounding box not detected,
- True Negative (TN): corrected misdetection.

In practical applications, the threshold for a detection being a *TP* is usually set to 0.5 or a higher value [40].

In the carried-out research, the following metrics used for assessing the quality of class predictions in object detection models were computed: precision, recall and *F*1-score [41,42]. These metrics are described by the following Formulas (3)–(5):

(3) $$precision=\frac{TP}{TP+FP}\;$$

(4) $$recall=\frac{TP}{TP+FN}\;$$

(5) $$F1=2\times \frac{recall\times precision}{recall+precision}\;$$

The training process also calculates the value of mean average precision (*mAP*). This metric is based on the concept of average precision (*AP*) of true positive detections for the *IoU* = 0.5. The *mAP* is a mean value of *AP* calculated for all the object classes which are defined for detection. In this paper, the YOLOv4 model has only one class to detect—pedestrian. That is why the value of *mAP* is equal to *AP*.

### 5.3. Training

For the deep neural network training process, the environment called Darknet was selected [42], which is an open-source neural network framework written in C programming language. As described in Section 3, the YOLOv4 method was chosen to train the pedestrian detection from the generated range images. To speed up the training, in the course of the carried-out research, the method called transfer learning was used. This method allows us to use an already-trained model and retrain only a part of the network layers to detect a specific object from a new custom dataset. In our experiments, the pre-trained weights, yolov4.conv.137 [43], have been applied.

The main goal is to answer the question of whether the synthetically generated data can be used to increase the performance of the pedestrian detector. That is why the first step was to select the validation dataset for all next training experiments. For this purpose, 600 frames from the Waymo dataset were chosen. To compare the performance of the detector trained on the synthetic data, firstly, the real data detector was trained. A total of 2.5 k frames were chosen to train the real data detector. The next step consisted in training the synthetic data detector. For this purpose, 2.5 k frames generated from Carla were also used. To fairly compare the detector performance, a dataset of the same size was used. The last step was to train the detector again, but with the use of all prepared data, so around 5 k frames, including a 50/50 proportion of the synthetic to real data. Since the performance of the detector trained with the mixed data was better than in case of the detector trained on real data, we extended the real dataset up to 5 k frames, the same size as for the mixed data.

The last training experiment was again performed with the use of the mixed data, but this time the dataset included 6.5 k of real data and 3.5 k of synthetic data. In this manner, five pedestrian detectors were trained with the use of real, synthetic and mixed datasets in different proportions.

**Figure 11:**
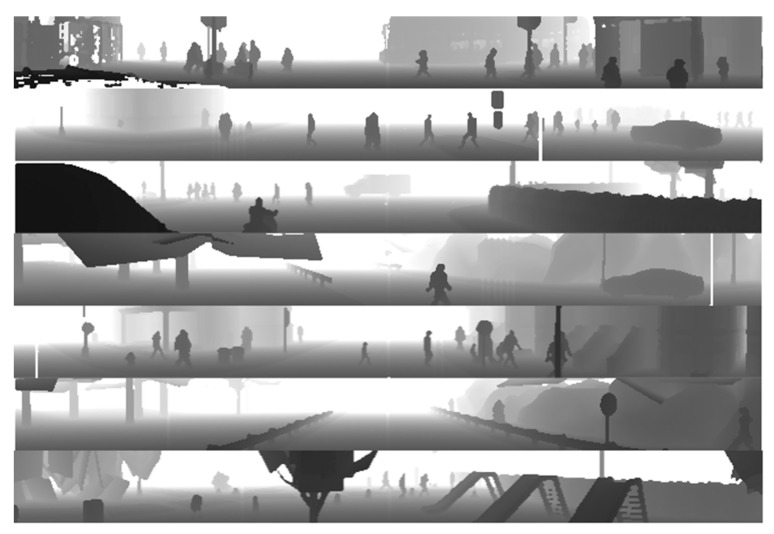
Example frames generated for training. Real range image from the Waymo dataset is the first one (at the top). All the next images are the synthetic data from the Carla simulator used for training.

The obtained results and a comparison of the trained model’s performance are described in the Section 6. Figure 11 depicts the example input frames from the real and synthetic data prepared for training.

## 6. Results

To compare the performance of each detector, the evaluation metrics, as described in Section 5.2, were calculated. Results of three detectors trained on the smaller datasets (real, synthetic and mixed data) are shown in Table 3.

**Figure 12:**
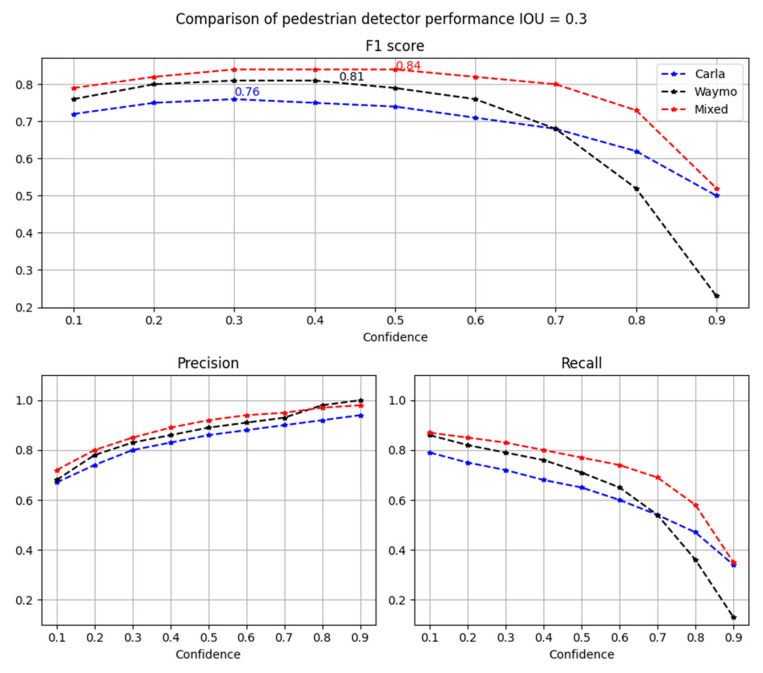
Comparison of pedestrian detector performance for *IoU* = 0.3.

To better visualize the differences between detectors, Figure 12 depicts the evaluation metrics as a function of the confidence threshold.

**Figure 13:**
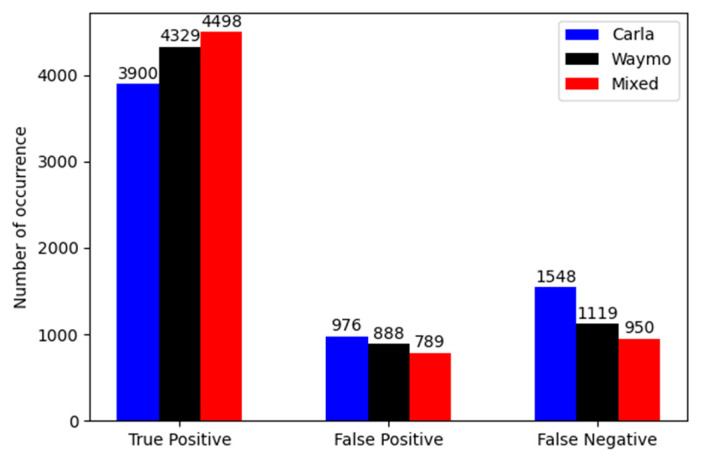
Number of true positives, false positives and false negatives for the synthetic, real and mixed dataset detectors. Metrics calculated for *IoU* = 0.3 and confidence threshold = 0.3.

Results depicted in Figure 12 show that the dataset which includes only synthetic data has the lowest performance. In comparison to the real data, it has a five percent lower *F*1-score. The situation is opposite for the mixed dataset. This detector shows an increase in performance by three percent of the *F*1-score in comparison to the real Waymo dataset. It is important to mark that all three models were validated on the same real data from the Waymo dataset. Figure 13 shows the number of true positives, false positives and false negatives for all three detectors.

The mixed dataset is twice as big as the real Waymo dataset, which is why following experiments with an extended real dataset were performed. Similar to the previous examples, mixed datasets with extended real data were also trained and compared. The performance of detectors trained with the extended datasets is shown in Table 4.

**Figure 14:**
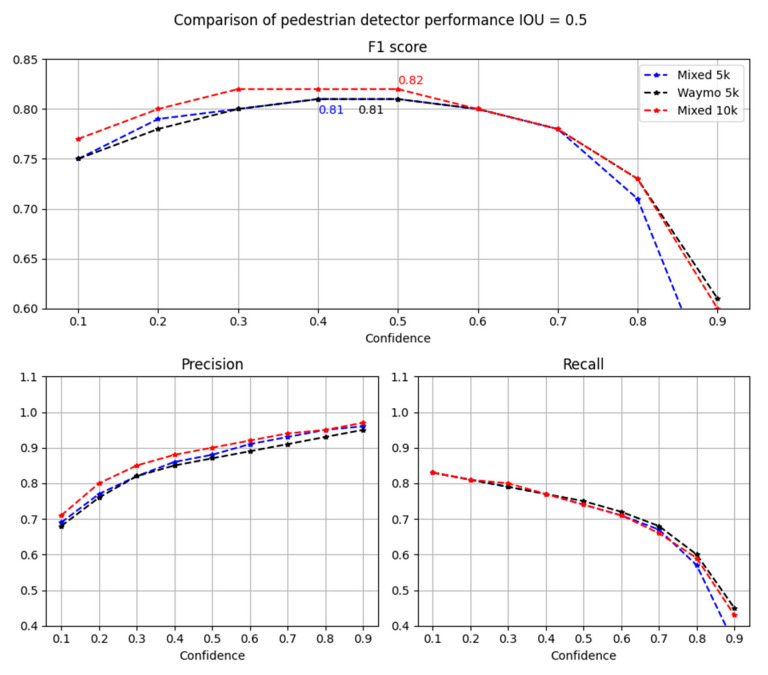
Comparison of pedestrian detectors’ performance trained on extended datasets (*IoU* = 0.5).

**Figure 15:**
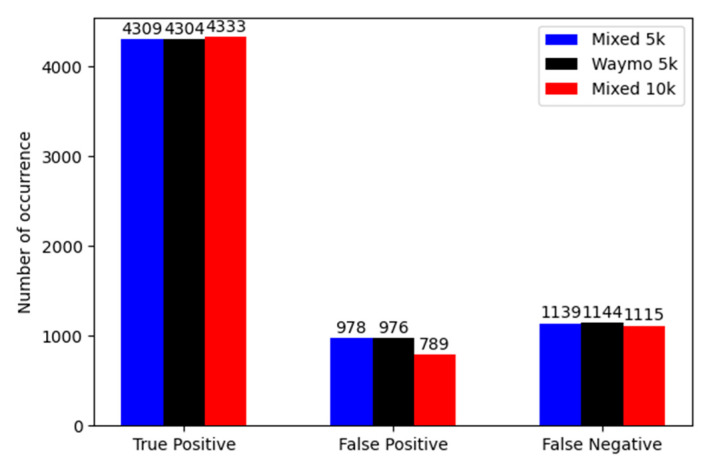
Number of true positives, false positives and false negatives for extended real and mixed dataset detectors. Metrics calculated for *IoU* = 0.5 and confidence threshold = 0.3.

In Figure 14, the results obtained for the extended datasets as a function of the confidence threshold are shown. Real and smaller mixed datasets have almost the same performance, but the extended mixed dataset shows a slight improvement. This small difference is visible on the bar graph of true positives, false positives and false negatives for the considered detectors (Figure 15).

In Figure 15, it is clearly visible that the bigger mixed dataset is slightly better in terms of false positive detections. Still, the experiment with extended datasets shows that around 35% of synthetic data in the whole dataset can increase the performance of the pedestrian detector. It is also promising that the same-sized dataset, including half of the data synthetically generated, has almost the same performance as the one trained with twice as much real data.

### 6.1. Inference Examples

**Figure 16:**
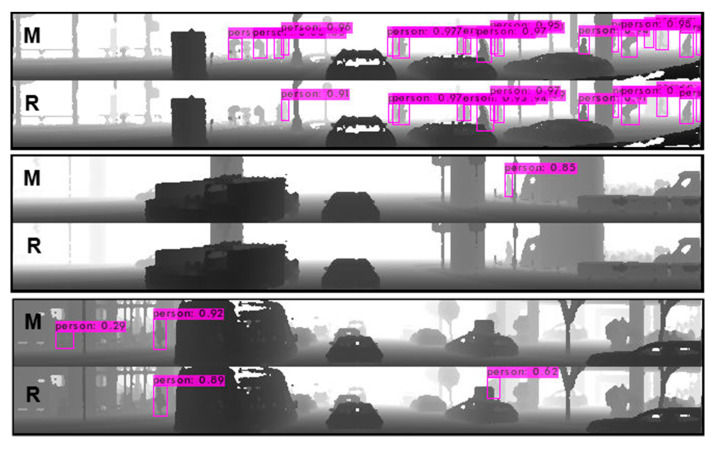
Three examples of inference showing differences between trained models (M—mixed dataset, R—real data only).

Examples showing the difference between pedestrian detection with the application of the detectors trained on the mixed and real data are depicted in Figure 16.

It is clearly visible (the first two pairs of pictures) that the model trained on the mixed dataset provides slightly better detection results. There are a few additional correct detections, but in the last example, for both models, there are two incorrect detections. What is interesting is that both incorrect detections are totally on the other side of the sensor’s view.

### 6.2. Discussion

In this paper, we presented the whole process of the LiDAR sensor synthetic data generation with the use of the Carla simulator. Such synthetically generated data were successfully used for creating the augmented Waymo dataset, in order to improve the performance of pedestrian detection. The presented method for synthetic data generation requires a PC equipped with a graphics card. After a few hours of driving in simulation, thousands of sensor frames are generated and saved. The most challenging task for this process is to provide a vast number of diverse scenarios to fit into the real dataset. In such a simulation, the easiest manner of proceeding is to prepare thousands of similar or identical frames, which are not useful in terms of machine learning. That is why most of the time spent on data generation was devoted to scenario preparation, putting pedestrians in the specified places and introducing realistic obstacles (such as parking meters, etc.) on the sidewalk. Simulation also has its limitations: at the moment, Carla provides only a few maps to drive, which means that the variety of scenarios to be prepared is limited. Generally, the generation process proposed in this paper is in the early stage of technical advancement. It happens that a part of the point cloud is not properly buffered, causing a drop in information. The technical issues are visible on the examples provided in Figs. 6 and 11. Unrealistic vertical lines appear on the frames. This is one of the differences between the real and synthetic data generated with the proposed method. Those vertical lines create unrealistic gaps in the range image which do not appear in the real dataset. The buffering algorithm somehow skips the last degree of the 360-degree view of the point cloud. Due to the rotational characteristic of the data generation, if this end of the view is on the 180-degree front view, it is processed and visible on the range image created from the point cloud. Nevertheless, to fit the synthetic range images into the shape and quality of the Waymo open dataset images, a median blur filter has to be applied. This filtering on range images makes the vertical lines less visible, so this difference is less important in the case of the pedestrian detector performance.

**Figure 17:**
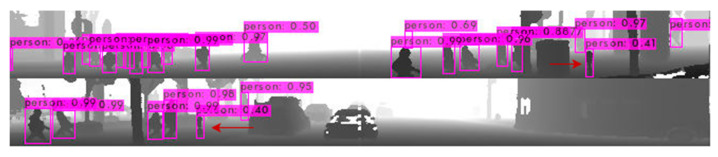
Examples of incorrect detections of pole-like objects as pedestrians.

To achieve a high performance level with the synthetic data, multiple models were trained. One example of improvements made to the dataset was the creation of the special scenarios with a variety of “pole-like” objects standing on the sidewalk. We found that the model trained on the synthetic dataset more likely detects parking meters, poles or small trees as pedestrians. Figure 17 depicts two examples of such incorrect detections.

**Figure 18:**
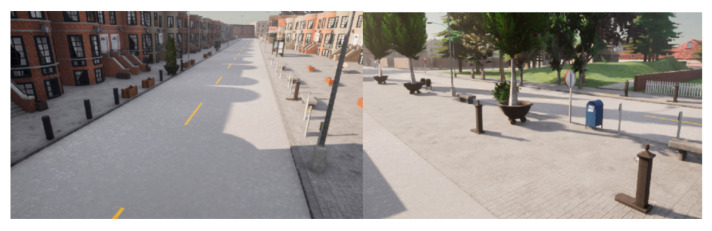
Example scenarios prepared with additional pole-like objects.

We decided to extend the synthetic dataset with the scenarios including a lot of those types of objects. To do this, we added pole-like objects into some maps, and then used those scenarios to save synthetic LiDAR data from it. Examples are shown in Figure 18.

Not only the scenarios prepared for data generation have a high impact on the detector performance. The positioning of the LiDAR sensor on the vehicle is also very important. First trainings with the use of the synthetic dataset had a much lower performance that the detectors trained on the real dataset, due to the incorrect positioning of the sensor on the vehicle. Positioning changes the perspective of the sensor view, so the objects such as pedestrians are a bit different in size and shape in the range images, which leads to a significant drop in accuracy, if the detector is evaluated on “different” real data.

In the course of the research, for validation purposes, 600 frames including data saved on a rainy day were selected. Rain itself has proven not to be a big deal, since its effect is not visible on the range images generated from the LiDAR point cloud, but its influence is visible directly on pedestrians. People on the road are carrying umbrellas (top picture in Figure 16). This totally standard situation cannot be simulated in Carla at the moment, at least not without heavy modification in the simulator. None of the synthetic data generated from the Carla simulator include pedestrians with additional attributes such as an umbrella, guitar, backpack or something else. This issue has a big impact on the performance of detectors trained with the use of synthetic data only.

All mentioned problems are technical ones and there are possibilities to fix them. The most important fact is that, even with such limitations, synthetically generated data are good enough to outperform the detector trained on real data only. Therefore, the methods proposed in this paper might be useful in autonomous vehicle applications.

## 7. Conclusions

The importance of the pedestrian detection problem has grown in recent years due to the increasing interest in the development of autonomous driving systems for vehicles. The evolution of pedestrian detection methods reported in the literature is directly related to the history of development of deep learning-based methods. The invention of the single-stage YOLO and its later versions can be treated as a turning point in its history, which opened the way to the development of reliable, automated sensor-based systems to detect pedestrians in autonomous vehicle applications.

Deep learning methods require a vast amount of data for training to reach a satisfactory level of performance. In order to prepare the vast datasets, a lot of human work to label the object of interest in the data is required. Even worse, the technical advancement of the sensors used for autonomous applications is rapidly growing. Most of the already labeled datasets is unusable due to differences between new and old sensor data generation and characteristics. Therefore, the process of data gathering and labeling is time- consuming and costly, without proof that this work can be used in future applications. That is why a lot of researchers work on the solution to the problem of providing synthetically generated datasets, which can be used for augmenting or even replacing the real dataset without differences in the performance of the algorithms. It is exciting that we would be able to use only synthetic data for the purpose of the early stages of development of autonomous systems to validate them without any risks of driving on public roads. Experiments conducted in this paper showed that, with the use of synthetic data, it is possible to evaluate developed algorithms or real data due to a small gap between real and synthetic data. This type of algorithm prototyping can significantly reduce the cost of validation of new methods or algorithms. Additionally, it is easier to validate the scenarios, which does not commonly happen on public roads.

The main goal of this paper was to answer the question of whether it is possible to improve the performance of pedestrian detection with the LiDAR sensor with the use of synthetically generated data. The experiment results provided in the paper show that the answer to this question is, yes, it is possible to improve deep network models by mixing the real and synthetic data for training. Moreover, the model trained on the mixed data outperforms the state-of-the-art pedestrian detectors developed by the research in [10,44]. The results also prove that a novel method for automatic labeling with the use of the semantic segmentation lidar can be successfully used to generate high-quality auto-labeled synthetic datasets. In the future, we plan to improve the data generation method and validate the synthetic data on other types of objects. Additionally, we want to improve the simulation to create more diverse and realistic road scenarios.

## Figures and Tables

**Table 1:** Simulated and Waymo top long-range LiDAR parameters [3].

Parameter	Lidar Simulation	Waymo Top Lidar
Number of channels	64	64
Measurement range [m]	75	75
Vertical field of view [˚]	+2.4/−17.6	+2.4/−17.6
Horizon field of view [˚]	360	360
Angular resolution horizontal [˚]	0.1358	0.1358
Rotation rate [Hz]	5	-
Points per second	0.848 M	-

**Table 2:** Additional sensor model parameters with description.

Parameter	Set Value	Description
Atmosphere attenuation rate	0.03	Coefficient that measures the LiDAR intensity loss per meter.
Dropoff general rate	0.35	General proportion of points that are randomly dropped.
Dropoff intensity limit	0.80	For the intensity-based drop-off, the threshold intensity value above which no points are dropped.
Dropoff zero intensity	0.40	For the intensity-based drop-off, the probability of each point with zero intensity being dropped.
Noise standard deviation [m]	0.02	Standard deviation of the noise model to disturb each point along the vector of its raycast.

**Table 3:** Comparison of pedestrian detector performance trained on the real, synthetic and mix data. Values were calculated for *IoU* = 0.3.

Dataset	Data Size	*F*1-Score	Precision	Recall
Synthetic Carla	2.5 k	0.76	0.8	0.72
Real Waymo	2.5 k	0.81	0.86	0.76
Mixed synthetic and real	5 k (2.5 k/2.5 k)	**0.84**	**0.89**	**0.8**

**Table 4:** Comparison of pedestrian detectors’ performance trained on real, synthetic and mixed data. Metric values were calculated for *IoU* = 0.5.

Dataset	Data Size	*F*1-Score	Precision	Recall
Mixed synthetic and real	5 k (50/50)	0.81	0.86	0.77
Extended real Waymo	5 K	0.81	0.85	0.77
Extended mixed	10 k (35/65)	**0.82**	**0.88**	**0.77**

## Data Availability

Not applicable.
